# Repeatability of corneal curvature with the MS-39 anterior segment tomographer in a cataractous population

**DOI:** 10.1371/journal.pone.0327863

**Published:** 2025-07-18

**Authors:** Achim Langenbucher, Nóra Szentmáry, Alan Cayless, Muntadher Al Karam, Theo G. Seiler, Jascha Wendelstein

**Affiliations:** 1 Department of Experimental Ophthalmology, Saarland University, Homburg/Saar, Germany; 2 Dr. Rolf M. Schwiete Center for Limbal Stem Cell and Aniridia Research, Saarland University, Homburg/Saar, Germany; 3 Department of Ophthalmology, Semmelweis-University, Budapest, Hungary; 4 School of Physical Sciences, The Open University, Milton Keynes, United Kingdom; 5 Institute for Refractive- and Ophthalmic Surgery (IROC), Zurich, Switzerland; 6 Department of Ophthalmology, Universitätsspital Zürich, Zurich, Switzerland; 7 Universitätsklinik für Augenheilkunde, Inselspital Bern, Bern, Switzerland; 8 Department of Ophthalmology, Ludwig-Maximilians-University, Munich, Germany; Massachusetts General Hospital, UNITED STATES OF AMERICA

## Abstract

**Purpose:**

The purpose of this study was to investigate the repeatability of optical coherence tomography in measuring radius of curvature (ROC) and asphericity (Q) of corneal epithelium, stroma and endothelium in a large patient cohort.

**Methods:**

In this retrospective non-randomized cross sectional single-center study we evaluated a dataset containing 600 anterior segment optical coherence tomography measurements from 200 eyes (3 repeat measurements each) taken prior to cataract surgery. Measurements were exported containing height map data for the epithelium, stroma and endothelium surface. Model surfaces (floating sphere in a 3 (BFS3) and 6 mm zone (BFS6) and conic surface in a 3 (Conoid3) and 6 mm zone (Conoid6)) were fitted using nonlinear iterative optimization minimizing the height difference between measurement and model. The mean (MEAN) and standard deviations (SD) for each sequence of measurements were derived and analyzed.

**Results:**

MEAN ROC for epithelium was 7.68/7.72/7.67/7.67 mm with BFS3/BFS6/Conoid3/Conoid6 and Q was −0.16/-0.19 with Conoid3/Conoid6. Corresponding values for stroma were 7.62/7.64/7.55/7.58 mm and −0.38/-0.24, and for endothelium they were 6.43/6.49/6.40/6.37 mm and −0.29/-0.33. Variations of the 3 repeat measurement ranged between 7–19/18–31/21–32 micron for epithelium/stroma/endothelium, and variation in Q was 0.14/0.20/0.14 with Conoid3 and 0.03/0.05/0.06 with Conoid6.

**Conclusions:**

Fitting floating sphere or conoid model surfaces to the map data seems to be a very robust and reliable method for extracting ROC and asphericity from all data within a region of interest. For extraction of asphericity, the 6 mm zone should be used due to fewer variations.

## Background

Intraocular lens (IOL) power calculation requires reliable data on corneal curvature. In addition to uncertainties in IOL power labelling, keratometry is known to be the most critical input parameter limiting the accuracy of lens power calculation and prediction of postoperative refraction after cataract surgery, followed by axial length measurement [1,2]. In a routine setup, we use (automated) keratometry from a modern optical biometer. These typically evaluate the position of 2 or more keratometer markers in at least the steep and flat meridians, and sometimes in more than 2 meridians [1–3]. In some modern biometers (automated) keratometry is replaced by Placido topography, Scheimpflug slit projection imaging, or optical coherence tomography (OCT), reading out areal data from the entire corneal front surface or the corneal front and back surface [4,5]. Those instruments have the capability to consider data derived from thousands of measurement points on the cornea, promising more robust measures especially in corneas with local surface irregularities or tear film pathologies [3,6]. Even where topographic or tomographic data are available, some biometers simulate the strategy of manual keratometry by analyzing corneal curvature at 2 distinct locations in the mid periphery 1.0 to 1.6 mm away from the corneal apex in the 2 cardinal meridians by using ‘simulated keratometry’ (SimK) values for lens power calculation. Additionally, most of the IOL power calculation formulae are restricted to working with data derived with a keratometer either in mm radius of curvature or converted to corneal power using a keratometer index for conversion. However, we know that keratometric data are not always representative for corneal power to be used in IOL power calculation, especially after refractive laser vision correction (LVC) procedures at the cornea (for example PRK or LASIK), radial keratotomies, or (penetrating or lamellar) keratoplasty, or with corneal pathologies such as ectatic diseases. These situations may exhibit unusual anterior/posterior curvature ratios [7], and we should also keep in mind that the cornea itself is not a monolayer structure with a homogeneous refractive index. The corneal epithelium shows a higher refractive index compared to the corneal stroma. This implies that we should consider the cornea as a dual layer structure with 3 refractive surfaces [8,9] to account for potential inhomogeneity in the epithelial thickness.

Modern high-resolution OCT devices for the anterior eye segment offer an excellent axial resolution [10–12], sufficient to separate the epithelial layer from the underlying stroma. For example, the Casia 2 from Tomey, the Anterion from Heidelberg Engineering, or the MS-39 from CSO all offer software tools with registration of the corneal front surface (epithelium), corneal stroma and corneal back surface (endothelium) [11,13–15]. The corresponding measurement data could be exported as map data (point cloud data) in CSV or Excel format for postprocessing in any consumer software. Making use of the refractive indices of the corneal epithelium and stroma as derived from the literature, the best focus of the cornea or corneal power could be calculated using raytracing strategies. For that purpose, a model surface has to be fitted to the 3D map data giving us the curvature of the cornea [2,6]. Restricting the analysis to rotational symmetry (i.e., nontoric IOLs) we could use a floating best fit sphere (BFS) or a floating conic (Conoid) surface. Such simple model surfaces could be defined using weighted or unweighted least squares fits or using iterative nonlinear fit techniques which have the benefit of easily extracted benchmarks and ranges for the parameters. The outputs of the BFS are the radius of curvature (ROC) and the apex position, and for the Conoid the central ROC, the asphericity and the apex position. Paraxial calculations can be used with the ROC and axial position of the corneal front surface, stromal surface and back surface, and raytracing could be used where the ROC, asphericity and the axial and lateral apex position are available, in both cases to determine the focus position or corneal power referenced, for example, to the corneal front apex plane. This could then be used in any IOL calculation software [8,9]. In contrast, considering the general case of corneal surfaces without rotational symmetry we could fit various surfaces such as biconic surfaces or fringe Zernike surfaces, and based on those surface models we could extract the best focus or corneal power with more advanced raytracing techniques. However, we should be cautious not to over-determine the surface with unnecessary additional parameters since, as a general rule of thumb, the uncertainty of the parameters tends to increase with the complexity of the model surface [2,6].

The **purpose of the present study** was

- to develop a strategy for reading and processing height map data of the corneal front surface, stromal surface and back surface exported from the MS-39 anterior segment OCT device,
- to implement robust fitting algorithms to fit a floating best fit sphere or a floating conic surface to the map data within a circular region of interest and to extract the characteristic surface descriptors from that surface model, and
- to show the clinical applicability of this technique based on a dataset containing 3 repeat measurements made with the MS-39 in a population prior to cataract surgery to show the repeatability of the extracted surface characteristics in a situation where reliable corneal power data are mandatory.

## Methods

### Dataset for our evaluation

A dataset containing 3 repeat measurements for each of 206 eyes of 103 patients (in total N = 618 measurements) taken prior to cataract surgery scheduled for implantation of a non-toric intraocular lens and without a history of eye surgery or corneal pathologies was considered in this study. All measurements were performed at the Institute for Refractive- and Ophthalmic Surgery (IROC) (Zurich, Switzerland) between June 2023 and December 2024 with the MS-39 anterior segment optical coherence tomography device (CSO, Scandicci, Italy). Each sequence of repeat measurements was completed within 10 min in each eye.

The measurement data were anonymized at source and transferred to.CSV map files using the software module for batch data export. For each measurement a separate CSV file was generated by the MS-39 software containing relevant patient data such as patient ID, the laterality (left or right eye), date of birth, sex (male or female), examination date and time, and map data with height data for the corneal epithelium, stroma and endothelium. Map data were organized in cylindrical coordinates with 256 meridians in 31 concentric rings with ring spacing of 0.2 mm (range from 0.0 to 6.0 mm). Invalid or unreliable data points within the map were indicated by a value of −1000.

The local ethics committee (IRB) has provided a waiver for this study (Ärztekammer des Saarlandes, 157/21), as all data processed in this study were already anonymized at source before being transferred to us for processing. This precludes any back-tracing of identity, and therefore informed consent of the patients was not necessary. Data tables were reduced to the relevant parameters required for our data analysis, consisting of the following measurements: patient ID and date of birth, exam date and time, the laterality (left or right eye), and height data for the corneal epithelium, stroma and endothelium within the central 6 mm zone. The data were transferred to Matlab (Matlab 2024a, MathWorks, Natick, USA) for further processing.

### Data pre-processing in Matlab

Patient ages were calculated from patient date of birth and examination date. The following model surfaces were fitted to the map data of corneal epithelium, stroma and endothelium: A) a floating best fit sphere within a region of interest (ROI) of 3 mm in diameter (BFS3) with 4 degrees of freedom: radius of curvature ROC and apex position in X (horizontally), Y (vertically) and Z (axially); B) a floating best fit sphere within a region of interest (ROI) of 6 mm in diameter (BFS6) with 4 degrees of freedom: radius of curvature ROC and apex position in X (horizontally), Y (vertically) and Z (axially); C) a floating conoid within a region of interest (ROI) of 3 mm in diameter (Conoid3) with 5 degrees of freedom: apical radius of curvature ROC, asphericity Q, and apex position in X (horizontally), Y (vertically) and Z (axially); D) a floating conoid within a region of interest (ROI) of 6 mm in diameter (Conoid6) with 5 degrees of freedom: apical radius of curvature ROC, asphericity Q, and apex position in X (horizontally), Y (vertically) and Z (axially). The surface fit was performed using nonlinear iterative optimization techniques (sequential quadratic programming, SQP algorithm), minimizing the root-mean-squared fit error in terms of the height difference between the measurement height data and the height data of the surface model. The resulting parameters were specified in terms of the model (either BFS3_, BFS6_, Conoid3_ or Conoid6_), the surface (epithelium (Epi), Stroma (Stroma) or endothelium (Endo)) and the indicator for the fit parameter (either R for ROC, Q for asphericity, X or Y for the lateral displacement, or Z for the axial displacement of the model surface apex). Corneal thickness was extracted from the differences in the Z positions of the model surface apices: epithelial thickness was derived from (.)StromaZ – (.)EpiZ, stroma thickness from (.)EndoZ – (.)StromaZ, and total corneal thickness from (.)EndoZ – (.)EpiZ.

In the next step we derived the mean values for all (.)R, (.)Q, (.)X, (.)Y and (.)Z parameters for the sequence of 3 repeat measurements (indicated by (.)m) and the deviation of all repeat measurements from the mean of the 3 repeat measurements (indicated by (.)d).

### Data processing in Matlab and statistics

The explorative statistics for the (.)m (per eye) and (.)d values of (.)R, (.)Q, (.)X, (.)Y and (.)Z (per measurement) is summarized in tables in terms of arithmetic mean, standard deviation, median, and the lower and upper boundaries of the 95% confidence interval (2.5% and 97.5% quantiles). For the deviations of the repeat measurements from their mean value, the mean (which equals zero) is not shown, but instead the repeatability coefficient RC = 2.77·standard deviation is listed. For visualization the distributions for (.)m and (.)d values of (.)R and (.)Q are shown using boxplots. The boxes refer to the interquartile range, and the whiskers to the 95% confidence interval. For the visualization of the 3D apex position vectors ((.)X, (.)Y and (.)Z), boxplots are used to display the distributions of (.)Z and polar scatterplots to display the distributions of the lateral apex position vectors (.)X and (.)Y. The 2D apex position vectors ((.)X and (.)Y) were analyzed for bivariate normality using the Henze-Zirkler test [16]. In cases of bivariate normality we calculated the centroids and the parametric error ellipses to display the 95% confidence ellipse for the scatter, and in cases of non-normality we calculated the nonparametric medoids [17–19] and used iterative convex hull stripping techniques to display the 95% confidence regions [20]. Since we expected mirror symmetry of the lateral 2D apex position vectors ((.)X and (.)Y) with respect to the vertical (facial) axis we separated left and right eyes for the polar scatterplots.

## Results

The dataset originally transferred to us contained N = 618 MS-39 examinations of the N = 206 eyes of N = 103 patients. After considering the selection criteria, a dataset with N = 600 measurements (N = 200 eyes) of N = 100 patients was selected for our analysis (100 right and 100 left eyes of 57 female and 43 male patients). Three patients were removed from the dataset due to a history of LVC. The mean age of the patients was 67.2 ± 9.7 years (median 68.3 years, 95% confidence interval from 52 to 84 years).

The upper block of Table 1 shows the mean values of the 3 repeat measurements in terms of radius of curvature data ((.)R) and asphericity data ((.)Q) for the BFS3, BFS6, Conoid3 and Conoid6 surface fit models and the epithelium, stroma, and endothelium surface. The lower block shows the deviations of the 3 repeat measurements from the corresponding mean values.

**Table 1:** Upper block: explorative data for the mean values of radius of curvature and asphericity for the 4 surface models (floating best fit sphere in a 3 mm (BFS3) and a 6 mm (BFS6) zone, Conoid in a 3 mm (Conoid3) and a 6 mm (Conoid6) zone) and for the 3 surfaces (epithelium, stroma and endothelium) as measured by the MS-39 anterior segment tomographer. Lower block: explorative data of the corresponding deviations of the 3 repeat measurements from their mean values. In the lower block the mean values are not shown as they all equal zero, but the repeatability coefficient RC is listed. SD refers to the standard deviation, and 2.5% quantile/ 97.5% quantile to the lower and upper boundaries of the 95% confidence interval.

Mean value of 3 repeat measurements (N = 200 eyes)							
Radius of curvature [mm], asphericity [1]		BFS3	BFS6	Conoid3		Conoid6	
		Radius of curvature in mm	Radius of curvature in mm	Radius of curvature in mm	Asphericity	Radius of curvature in mm	Asphericity
Epithelium	Mean	7.6814	7.7169	7.6680	−0.1629	7.6716	−0.1859
	SD	0.3223	0.3061	0.3293	0.2670	0.3304	0.1870
	Median	7.6659	7.7197	7.6696	−0.1855	7.6491	−0.1912
	2.5% quantile	7.0659	7.1687	7.0491	−0.7112	7.0454	−0.5538
	97.5% quantile	8.5016	8.3894	8.5189	0.4371	8.5534	0.2298
Stroma	Mean	7.6174	7.6438	7.5540	−0.3837	7.5817	−0.2375
	SD	0.3557	0.3261	0.3753	0.3359	0.3769	0.2582
	Median	7.6146	7.6465	7.5337	−0.43321	7.5554	−0.2225
	2.5% quantile	6.9539	7.0554	6.8608	−1.1311	6.9352	−0.8676
	97.5% quantile	8.4709	8.4002	8.4048	0.3988	8.5553	0.3205
Endothelium	Mean	6.4259	6.4892	6.4001	−0.2938	6.3687	−0.3298
	SD	0.3107	0.2683	0.3349	0.4276	0.3231	0.2162
	Median	6.4210	6.4861	6.4144	−0.2372	6.3634	−0.3383
	2.5% quantile	5.8624	5.9950	5.7624	−1.1187	5.8721	−0.8129
	97.5% quantile	7.0922	7.0679	7.1121	0.5379	7.0532	0.0985
Deviation of the 3 repeat measurement from the mean value (N = 600 measurements)							
Epithelium	SD	0.0110	0.0070	0.0194	0.1358	0.0111	0.0261
	RC	0.0303	0.0195	0.0538	0.3761	0.0307	0.0722
	Median	−0.0002	−0.0001	0.0003	0.0012	0.0001	−0.0002
	2.5% quantile	−0.0231	−0.0133	−0.0435	−0.2478	−0.0240	−0.0459
	97.5% quantile	0.0218	0.0144	0.0389	0.2914	0.0247	0.0499
Stroma	SD	0.0212	0.0176	0.0310	0.2038	0.0289	0.0522
	RC	0.0586	0.0488	0.0860	0.5645	0.0802	0.1447
	Median	0.0001	−0.0001	0.0000	0.0000	0.0000	−00012
	2.5% quantile	−0.0299	−0.0178	−0.0642	−0.4423	−0.0325	−0.0843
	97.5% quantile	0.0310	0.0187	0.0682	0.5040	0.0354	0.0821
Endothelium	SD	0.0208	0.0198	0.0323	0.1419	0.0226	0.0583
	RC	0.0577	0.0548	0.0896	0.3930	0.0626	0.1617
	Median	0.0002	0.0002	0.0003	0.0007	0.0002	0.0004
	2.5% quantile	−0.0186	−0.0147	−0.0363	−0.2822	−0.0223	−0.0494
	97.5% quantile	0.0185	0.0157	0.0353	0.2741	0.0210	0.0550

The upper block of Table 2 shows the mean values of the 3 repeat measurements in terms of horizontal apex position (.)X, vertical apex position (.)Y and axial apex position (.)Z for the BFS3, BFS6, Conoid3 and Conoid6 surface fit models and the epithelium, stroma, and endothelium surface. The lower block shows the deviations of the 3 repeat measurements from the corresponding mean values.

**Table 2:** Upper block: explorative data for the mean values of X, Y and Z apex position of the fit models for the 4 surface models (floating best fit sphere in a 3 mm (BFS3) and a 6 mm (BFS6) zone, Conoid in a 3 mm (Conoid3) and a 6 mm (Conoid6) zone) and the 3 surfaces (epithelium, stroma and endothelium) as measured by the MS-39 anterior segment tomographer. X and Y refer to the horizontal and vertical displacements, and Z to an axial displacement. Lower block: explorative data of the corresponding deviations of the 3 repeat measurements from their mean values (shown in the upper block). In the lower block the mean values are not shown as they all equal zero, but the repeatability coefficient RC is listed. SD refers to the standard deviation, and 2.5% quantile/ 97.5% quantile to the lower and upper boundaries of the 95% confidence interval.

Mean value of 3 repeat measurements (N = 200 eyes)													
All data in mm		BFS3			BFS6			Conoid3			Conoid6		
		X	Y	Z	X	Y	Z	X	Y	Z	X	Y	Z
Epithelium	Mean	0.0003	0.0002	0.0000	0.0009	0.0026	0.0004	0.0003	0.0002	0.0000	0.0009	0.0026	0.0000
	SD	0.0045	0.0063	0.0001	0.0111	0.0165	0.0005	0.0045	0.0063	0.0001	0.0111	0.0166	0.0002
	Median	0.0000	0.0000	0.0000	0.0006	0.0009	0.0004	0.0000	0.0000	0.0000	0.0006	0.0009	0.0000
	2.5% quantile	−0.0064	−0.0108	−0.0001	−0.0187	−0.0192	−0.0008	−0.0064	−0.0108	−0.0001	−0.0187	−0.0192	−0.0002
	97.5% quantile	0.0073	0.0107	0.0003	ß-ß193	0.0378	0.0012	0.0073	0.0107	0.0002	0.0193	0.0377	0.0006
Stroma	Mean	0.0007	0.0012	0.0547	0.0027	0.0043	0.0551	0.0007	0.0012	0.0546	0.0027	0.0043	0.0546
	SD	0.0120	0.0132	0.0043	0.0127	0.0202	0.0043	0.0120	0.0133	0.0044	0.0127	0.0203	0.0044
	Median	0.0007	0.0012	0.0547	0.0021	0.0025	0.0549	0.0007	0.0012	0.0547	0.0021	0.0025	0.0545
	2.5% quantile	−0.0163	−0.0229	0.0475	−0.0187	−0.0237	0.0482	−0.0163	−0.0228	0.0474	−0.0187	−0.0237	0.0469
	97.5% quantile	0.0199	0.0296	0.0627	0.0275	0.0422	0.0638	0.0202	0.0295	0.0626	0.0277	0.0423	0.0626
Endothelium	Mean	−0.0037	−0.0456	0.5402	−0.0020	−0.0446	0.5415	−0.0037	−0.0457	0.5401	−0.0021	−0.0450	0.5400
	SD	0.0566	0.0412	0.0389	0.0518	0.0375	0.0390	0.0567	0.0414	0.0389	0.0523	0.0380	0.0390
	Median	0.0067	−0.0432	0.5386	0.0001	−0.0436	0.5398	0.0067	−0.0431	0.5385	−0.0003	−0.0438	0.5383
	2.5% quantile	−0.1054	−0.1208	0.4549	−0.0920	−0.1188	0.4557	−0.1058	−0.1238	0.4551	−0.0929	−0.1213	0.4547
	97.5% quantile	0.0836	0.0217	0.6110	0.0789	0.0314	0.6122	0.0841	0.0217	0.6111	0.0798	0.0316	0.6123
Deviation of the 3 repeat measurement from the mean value (N = 600 measurements)													
Epithelium	SD	0.0014	0.0010	0.0000	0.0020	0.0016	0.0001	0.0014	0.0010	0.0000	0.0020	0.0016	0.0001
	RC	0.0040	0.0029	0.0001	0.0055	0.0046	0.0002	0.0040	0.0028	0.0001	0.0055	0.0046	0.0001
	Median	0.0000	0.0000	0.0000	0.0000	0.0000	0.0000	0.0000	0.0000	0.0000	0.0000	0.0000	0.0000
	2.5% quantile	−0.0026	−0.0018	−0.0001	−0.0040	−0.0028	−0.0001	−0.0026	−0.0018	−0.0001	−0.0040	−0.0028	−0.0001
	97.5% quantile	0.0024	0.0019	0.0001	0.0036	0.0029	0.0001	0.0024	0.0019	0.0001	0.0036	0.0029	0.0001
Stroma	SD	0.0064	0.0049	0.0005	0.0034	0.0035	0.0007	0.0065	0.0049	0.0005	0.0034	0.0035	0.0007
	RC	0.0179	0.0135	0.0015	0.0094	0.0096	0.0019	0.0179	0.0135	0.0014	0.0095	0.0096	0.0020
	Median	−0.0001	0.0000	0.0000	0.0000	0.0000	0.0000	−0.0001	0.0000	0.0000	0.0000	−0.0001	0.0000
	2.5% quantile	−0.0073	−0.0064	−0.0010	−0.0076	−0.0066	−0.0008	−0.0073	0.0064	−0.0009	−0.0076	−0.0066	−0.0010
	97.5% quantile	0.0071	0.0071	0.0010	0.0075	0.0076	0.0010	0.0071	0.0071	0.0010	0.0075	0.0076	0.0009
Endothelium	SD	0.0087	0.0077	0.0008	0.0040	0.0070	0.0012	0.0085	0.0076	0.0008	0.0041	0.0071	0.0011
	RC	0.0242	0.0213	0.0022	0.0110	0.0194	0.0034	0.0237	0.0212	0.0022	0.0114	0.0196	0.0031
	Median	−0.0001	−0.0001	0.0000	0.0000	−0.0001	0.0000	−0.0001	−0.0001	0.0000	0.0000	−0.0001	0.0000
	2.5% quantile	−0.0070	−0.0086	−0.0012	−0.0076	−0.0097	−0.0012	−0.0069	−0.0086	−0.0011	−0.0076	−0.0098	−0.0012
	97.5% quantile	0.0070	0.0105	0.0013	0.0072	0.0102	0.0013	0.0071	0.0104	0.0012	0.0072	0.0102	0.0013

**Fig 1:**
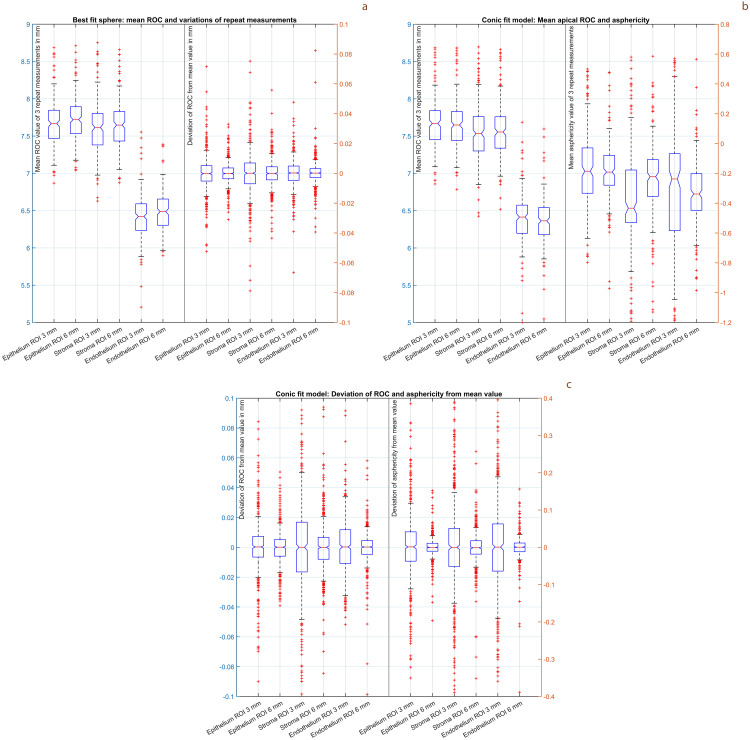
Boxplots showing the mean values of the 3 repeat measurements and the deviations of the 3 repeat measurements from the corresponding mean values for the radius of curvature ROC and asphericity for the 3 corneal surfaces (epithelium, stroma and endothelium) and for the 4 surface fit models (floating sphere fitted within a region of interest (ROI) 3 mm (BFS3) and 6 mm (BFS6) and a conoid fitted within a region of interest 3 mm (Conoid3) and 6 mm (Conoid6)). **Subfigure 1a** displays the distributions of the ROC for BFS3 and BFS6, **subfigure 1b** displays the distributions of the mean ROC and asphericity for Conoid3 and Conoid6, and **subfigure 1c** displays the distribution of the deviation (of the repeat measurements from the mean) of the ROC and asphericity for Conoid3 and Conoid6. The scales on the left Y axis refer to the plots on the left side of the graph and the scales on the right Y axis to the plots on the right side of the graph.

Fig 1 displays the mean values of the 3 repeat measurements and the deviations of the repeat measurements from the mean values for the ROC and the asphericity. In **subfigure 1a** the mean values of the ROC for BFS3 and BFS6 are shown on the left side and the corresponding deviations of the 3 repeat measurements from the mean values are shown on the right side. In **subfigure 1b** the mean values of the ROC for Conoid3 and Conoid6 are shown on the left side and the mean values of the asphericities for Conoid3 and Conoid6 are shown on the right side. In **subfigure 1c** the deviations of the 3 repeat measurements from the mean values for ROC (left side) and asphericity (right side) are displayed for the Conoid3 and Conoid6 model surface.

**Fig 2:**
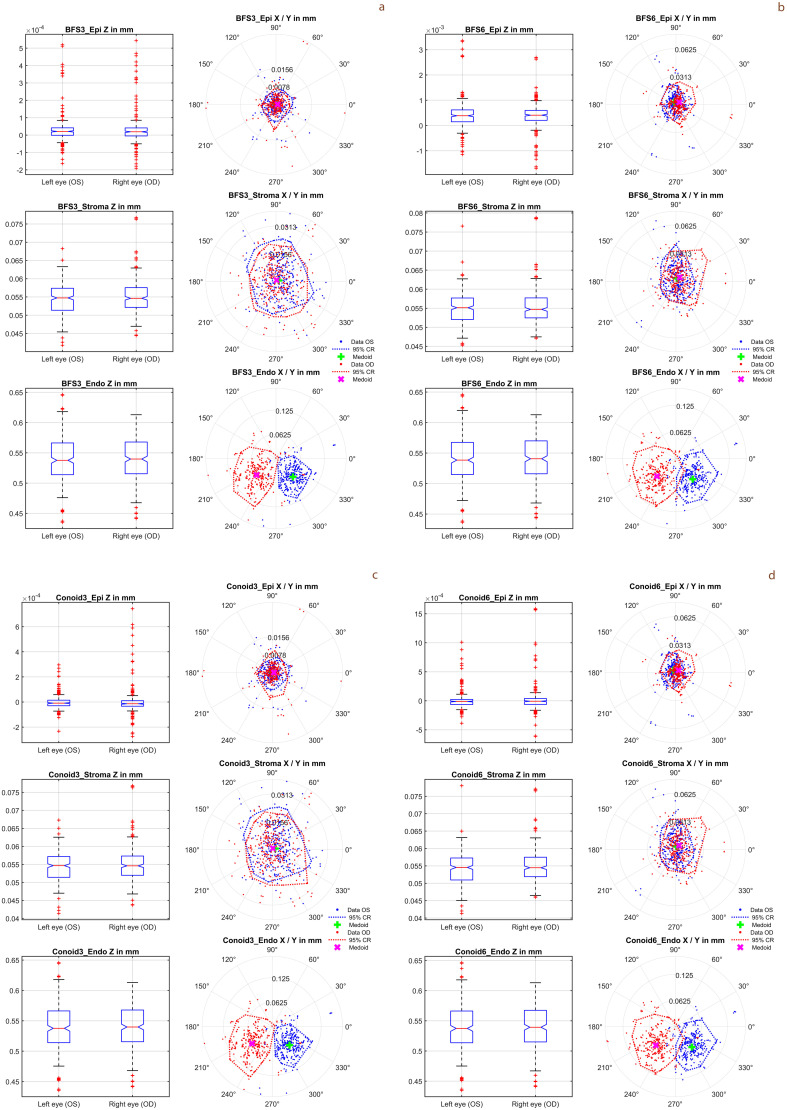
Distributions of the mean values of the apex position derived from the 3 repeat measurements for the corneal epithelium ((. )_Epi, upper graph), stroma ((.)_Stroma, middle graph), and endothelium ((.)_Endo, lower graph). X and Y (right hand plots) correspond to the horizontal and vertical displacements and Z to the axial displacement (boxplots on left). Note the differences in the vertical scales (Z) for each of the three left hand plots in each subfigure. Subfigure 2a represents the situation for the floating sphere fitted within a region of interest (ROI) of 3 mm, subfigure 2b shows the situation for the floating sphere fitted within a region of interest (ROI) of 6 mm, subfigure 2c shows the situation for the floating conoid fitted within a region of interest (ROI) of 3 mm, and subfigure 2d displays the situation for the floating conoid fitted within a region of interest (ROI) of 6 mm. Since none of the bivariate distributions of the lateral displacement showed normality, we displayed the medoids and the 95% confidence region (CR) instead of centroids and 95% confidence ellipses. The lower right graphs in all subfigures indicate that the apex position exhibits some mirror symmetry of left and right eyes with respect to the Y axis.

Fig 2 shows the mean values of the apex position derived from the 3 repeat measurements for the corneal epithelium (upper graph), stroma (middle graph), and endothelium (lower graph). **Subfigure 2a** represents the situation for BFS3, **subfigure 2b** the situation for BFS6, **subfigure 2c** the situation for Conoid3, and **subfigure 2d** the situation for Conoid6. The variations in the thickness of the epithelium, the stroma, and the entire cornea based on these surface fits can be derived from the Z positions displayed on the left graphs. For each of these subfigures, it can be seen from the locations of the medoids in the right graphs that the apex position, especially in the case of the surface fit to the endothelium (lower graph), exhibits some mirror symmetry with respect to the Y axis.

The explorative data for the thickness of the corneal epithelium, stroma, and the entire cornea derived from the surface fit with the 4 surface fit models BFS3, BFS6, Conoid3, and Conoid6 are shown in Table 3. The mean values of the 3 repeat measurements are listed in the upper part of the table, and the deviations of the 3 repeat measurements from the corresponding mean values are shown in the lower part.

**Table 3:** Thickness of the corneal epithelium (Epi), corneal stroma (Stroma), and total corneal thickness (Total) derived from the surface fit with a floating sphere within the 3 mm (BFS3) or 6 mm (BFS6) region of interest, or from a surface fit with a floating conoid within the 3 mm (Conoid3) or 6 mm (Conoid6) region of interest. The upper part of the table lists the mean values of the 3 repeat measurements, and the lower part lists the deviations of the 3 repeat measurements from the corresponding mean values. SD refers to the standard deviation, and 2.5% quantile/ 97.5% quantile to the lower and upper boundaries of the 95% confidence interval. In the lower block the mean values are not shown as they all equal zero, but the repeatability coefficient RC is listed.

Thickness in mm	BFS3			BFS6			Conoid3			Conoid6		
	Epi	Stroma	Total	Epi	Stroma	Total	Epi	Stroma	Total	Epi	Stroma	Total
Mean value of 3 repeat measurements (N = 200 eyes)												
Mean	0.0547	0.4855	0.5402	0.0547	0.4864	0.5411	0.0546	0.4856	0.5402	0.0546	0.4855	0.5400
SD	0.0044	0.0387	0.0389	0.0043	0.0388	0.0390	0.0044	0.0387	0.0389	0.0045	0.0388	0.0390
Median	0.0547	0.4834	0.5386	0.0546	0.4856	0.5396	0.0547	0.4834	0.5384	0.0545	0.4835	0.5384
2.5% quantile	0.0474	0.4083	0.4548	0.0479	0.4075	0.4551	0.0474	0.4086	0.4551	0.0469	0.4087	0.5459
97.5% quantile	0.0626	0.5534	0.6110	0.0633	0.5578	0.6117	0.0626	0.5537	0.6111	0.0627	0.5542	0.6123
Deviation of the 3 repeat measurement from the mean value (N = 600 measurements)												
SD	0.0005	0.0008	0.0008	0.0007	0.0009	0.0012	0.0005	0.0008	0.0008	0.0007	0.0008	0.0011
RC	0.0015	0.0022	0.0022	0.0019	0.0025	0.0034	0.0014	0.0022	0.0022	0.0020	0.0023	0.0031
Median	0.0000	0.0000	0.0000	0.0000	0.0000	0.0000	0.0000	0.0000	0.0000	0.0000	0.0000	0.0000
2.5% quantile	−0.0010	−0.0013	−0.0012	−0.0009	−0.0014	−0.0013	−0.0010	−0.0012	−0.0011	−0.0010	−0.0014	−0.0013
97.5% quantile	0.0010	0.0014	0.0013	0.0010	0.0014	0.0012	0.0010	0.0014	0.0012	0.0010	0.0015	0.0013

## Discussion

Anterior segment tomographers have developed significantly over the last 2 decades, and they are known to offer precise and highly reproducible data on the cornea, anterior chamber, and the crystalline or artificial lens. Several scientific studies have been performed to investigate the comparability, precision and repeatability of anterior segment tomographers [1,3–5,10,12,20–32]. Some newer optical biometers have adopted this idea and implement Scheimpflug or optical coherence tomography in contrast to classical keratometry to measure corneal shape and relevant metrics such as anterior chamber depth or central lens thickness [4,5]. In most clinical setups the ‘true value’ of the corneal power is not relevant, and ophthalmologists mainly document abnormalities of corneal curvature or monitor the time course for deciphering pathologies or follow-up. However, for lens power calculation prior to cataract surgery, we require the ‘true value’ of the corneal power [1,3]. With keratometry, corneal power is derived from corneal front-surface curvature using a keratometer index, but this keratometer index is based on specific model assumptions with some simplifications which may not be applicable to any specific keratometer. This means that keratometry does not represent corneal power in a general case. In certain cases, and especially after LVC or keratoplasty, or with ectatic diseases or corneal edema, these model assumptions are no longer valid. To avoid using a keratometer index, tomographers measure the corneal front and back surfaces, and clinicians could then use a thick lens model of the cornea to specify corneal power. Such a thick lens model is known from the literature to have great advantages over keratometry, especially in abnormal corneas. However, we know that the cornea is not a homogeneous monolayer, and that the refractive index of epithelium is systematically higher compared to the stroma. For more than 2 decades it has been known that complications such as epithelial hyperplasia after hyperopic LASIK or PRK could systematically affect corneal power and promote regression of the refraction towards the original hyperopic refraction before LVC. In simple terms this means that the donut ablation profile is ‘re-filled’ in part with epithelium, which acts as an additional epithelial minus lens on top of the stroma.

The most recent development in high-resolution anterior segment tomographers such as the Heidelberg Engineering Anterion or CSO MS-39 is the implementation of epithelial mapping [8–10,27]. The software modules identify and register the cornea with 3 refracting surfaces, and surface map data (point cloud data) are generated for direct access or for export in a public file format for post-processing.

In the present paper we used the exported surface height map data of the corneal epithelium, stroma and endothelium surface from a population scheduled for cataract surgery with implantation of a non-toric intraocular lens to fit characteristic model surfaces and to analyze the variability of repeat measurements. The variations in a sequence of repeat measurements could be caused by a number of factors, such as small fixation instabilities or variations of the visual axis due to pupil size variations, or due to changes in the tear film configurations. As examples, we used 4 model surfaces including a floating best fit sphere within central 3 and 6 mm zones and a floating conoid within central 3 and 6 mm zones. In contrast to ‘simulated keratometry’ measures which extract the surface curvature at distinct points similar to manual keratometry, we wanted to use a more robust extraction of curvature metrics from the entire region of interest (either 3 or 6 mm). Such rotationally symmetric model surfaces are sufficient for surface fitting when the analysis is restricted to implantations of non-toric lenses. However, this concept could easily be generalized to also account for corneal astigmatism by making use of non- rotationally symmetric surface models such as biconic surfaces or fringe Zernike surfaces [2,6,33]. The floating sphere surface models generate values for the radii of curvature and the apex positions of the 3 corneal surfaces, and the floating conoid additionally provides some information on the surface asphericity.

The dataset that we finally used for analysis contained measurement data from 200 eyes of 100 patients, with a sequence of 3 repeat measurements for each eye. After extracting the characteristic metrics from all 600 examinations, we calculated the mean value over the 3 repeat measurements as well as the parameter deviations of the 3 measurements from the mean value for each of the parameters. In that context, the standard deviation of the deviations from the mean value (.)d represents the within-subject standard deviation used as a characteristic repeatability metric. From the apex position of the 3 corneal surfaces the entire corneal thickness and the thickness of the epithelium and stroma were calculated as listed in Table 3.

For both the BFS3 and BFS6 (Fig 1a on the left side) and for the Conoid 3 and Conoid6 (Fig 1b on the left side) model fits, our results indicate that the radius of curvature of the epithelium is slightly larger compared to that of the stroma, and the radius of curvature of the endothelium is systematically lower. This is not surprising, if we consider the cornea as an onion-like structure in which the more posterior located surfaces are more curved than the more anterior surfaces. However, it was surprising to us that corneal asphericity values as characterized by the Conoid3 and Conoid6 model fits (as shown in Fig 1b on the right side) show a large variation in our population from −1.2 to 0.8. This means that considering the pupil size together with corneal asphericity could potentially improve the quality of intraocular lens power calculation in the future. However, what we learn from the deviation of the repeat measurements from the mean value (Fig 1a on the right side for BFS3 and BFS6 and Fig 1c for Conoid 3 and Conoid6), is that the model fit with a floating sphere or conoid seems to be very robust, with the variation of the radius of curvature in the 3 repeat measurements typically in a range of 10–20 microns for all fit models under test. However in contrast, especially for the 3 mm region of interest (Conoid3), the variation of the asphericity in the 3 repeat measurements (range 0.13 to 0.20) is systematically larger as compared to the corresponding values from the fit with Conoid6 (range 0.03 to 0.06). This means that if we are interested in the asphericity of any corneal surface, we should rely on larger regions of interest. This would be of particular relevance in cases with larger physiological pupil sizes.

From the boxplots on the left of Fig 2 it can be seen that the variation of the axial apex position is quite low for the epithelium, and systematically larger for the stroma and endothelium. The explanation is quite straightforward: because the origin of the internal MS-39 coordinate system matches the ‘highest point’ of the corneal epithelium, the variation of the apex in Z direction is low. However, as the epithelial and stromal thickness shows some variation in the population, the variations in the Z positions of the fit models for the stroma and endothelium increase. It might be interesting to note that with the BFS3 and BFS6 the Z position of the apex for the epithelium is slightly in the positive range. This is a consequence of the negative asphericity of the corneal front surface. With a spherical fit on this aspherical surface the apex shifts slightly upwards. From the graphs on the right of Fig 2, it can be seen that the medoids indicating the lateral displacement of the model surface apex are well centred for the corneal epithelium and the stroma, but we see a systematic shift of the apex for the corneal endothelium in the inferior and temporal directions, reflecting the tilt of the visual axis in a normal population. The lateral component of this shift shows some mirror symmetry between left and right eyes with respect to the facial axis.

It is surprising that the apex positions of the epithelium, stroma and endothelium derived from the BFS3, BFS6, Conoid3 and Conoid6 surface fits show excellent consistency over the 3 repeat measurements as shown in the lower part of Table 2. The within-subject standard deviation Sw for the axial (Z) and for both lateral components (X and Y) is in a range of up to 9 microns. This again confirms that the measurements are highly standardized and demonstrates that the nonlinear iterative surface fit strategy with floating spheres and conoids is quite robust.

However, the present study has some limitations: A) we used a single center dataset with repeat measurements from a MS-39 anterior segment tomographer. In a multicenter setup or with different anterior segment tomographers the results might differ. B) as the study was restricted to a sequence of 3 repeat measurements for each eye we were not able to derive the distribution of the variations for the repeat measurements. However, this information might be helpful in the future for implementing error propagation models. C) all measurements considered in this study are from a population scheduled for cataract surgery with an implantation of a non-toric intraocular lens (with low to moderate corneal astigmatism). Including repeat tomography measures from a young population and from patients with large or excessive corneal astigmatism results may differ, and for extraction of corneal astigmatism other fit models such as biconic or Zernike surfaces have to be used instead of floating spheres or conoids. D) In contrast to univariate statistics, for bivariate analysis there is no unique strategy for dealing with non-normality of data. In this paper we used medoids and confidence regions derived from iterative convex hull stripping, but other complimentary techniques might be used.

**In conclusion**, in the present study we investigated the repeatability of a modern anterior segment tomographer used for extracting the curvature and asphericity of the corneal epithelium, stroma and endothelium surface. Height map data exported with the standard software of the MS-39 were used to fit floating spheres and conoids, both within central regions of interest of 3 and 6 mm, and the mean values of 3 repeat measurements and deviations of the repeat measurements from the mean value were assessed. The results for the radius of curvature and for the asphericity were extracted and appear to be highly consistent between the 3 repeat measurements. Additional multicentric studies with larger sequences of repeat measurements, enlarged age and astigmatism range, and additional surface models which take into account any astigmatism of the 3 corneal surfaces, could help to generalize the results and establish dedicated error propagation models to better understand the impact of the corneal epithelium, stroma and endothelium surface and their measurement uncertainties on intraocular lens power calculations with intraocular lenses.

### Disclosure of potential conflict of interest

All authors certify that they have no affiliations with or involvement in any organization or entity with any financial interest (such as honoraria (except speaker fees as outlined below); educational grants; participation in speakers’ bureaus; membership, employment, consultancies, stock ownership, or other equity interest; and expert testimony or patent-licensing arrangements), or non-financial interest (such as personal or professional relationships, affiliations, knowledge or beliefs) in the subject matter or materials discussed in this manuscript.

Dr. Langenbucher reports speaker fees from Bausch & Lomb and Johnson & Johnson Vision outside the submitted work.

Dr. Szentmáry, Dr. Cayless, Dr. Al-Karam and Dr. Seiler do not report any financial interests.

Dr. Wendelstein reports speaker fees from Carl Zeiss Meditec AG, Rayner, Alcon and Johnson & Johnson Vision outside of the submitted work.
